# Detection of Circulating Tumour Cells from Blood of Breast Cancer Patients via RT-qPCR

**DOI:** 10.3390/cancers5041212

**Published:** 2013-09-25

**Authors:** Ulrich Andergassen, Alexandra C. Kölbl, Stefan Hutter, Klaus Friese, Udo Jeschke

**Affiliations:** Department of Obstetrics and Gynaecology, Ludwig Maximilians University of Munich, Munich, Maistrasse 11, D-80337 Munich, Germany; E-Mails: ulrich.andergassen@med.uni-muenchen.de (U.A.); alexandra.koelbl@med.uni-muenchen.de (A.C.K.); stefan.hutter@med.uni-muenchen.de (S.H.); klaus.friese@med.uni-muenchen.de (K.F.)

**Keywords:** circulating tumour cell (CTC), breast cancer, cytokeratin, marker gene, reverse transcriptase, qPCR

## Abstract

Breast cancer is still the most frequent cause of cancer-related death in women worldwide. Often death is not caused only by the primary tumour itself, but also by metastatic lesions. Today it is largely accepted, that these remote metastases arise out of cells, which detach from the primary tumour, enter circulation, settle down at secondary sites in the body and are called Circulating Tumour Cells (CTCs). The occurrence of such minimal residual diseases in the blood of breast cancer patients is mostly linked to a worse prognosis for therapy outcome and overall survival. Due to their very low frequency, the detection of CTCs is, still a technical challenge. RT-qPCR as a highly sensitive method could be an approach for CTC-detection from peripheral blood of breast cancer patients. This assumption is based on the fact that CTCs are of epithelial origin and therefore express a different gene panel than surrounding blood cells. For the technical approach it is necessary to identify appropriate marker genes and to correlate their gene expression levels to the number of tumour cells within a sample in an *in vitro* approach. After that, samples from adjuvant and metastatic patients can be analysed. This approach may lead to new concepts in diagnosis and treatment.

## 1. Breast Cancer

Breast cancer is the most common cancer in women worldwide. It makes up for 16% of all female cancers. One out of eight women is diagnosed with breast cancer during her lifetime. In 2004, 519,000 women died of breast cancer and its secondary effects [1]. Despite improving survival rates in the last 40 years, the median age of death is 68 years old [2]. A positive outcome due to improved cancer control strategies, early cancer detection and improved treatment has lead to three quarters of the patients survive for at least 10 years [3]. Therefore, the main reason for cancer-associated death is not the primary tumour, but rather the formation of remote metastases in other organs. According to current models, single cells dissolve from the primary tumour and are the basis of metastasis formation [4].

## 2. Circulating Tumour Cells

Detached tumour cells, which circulate through blood stream or lymphatic vessels are called circulating tumour cells (CTCs) [5]. These cells become disseminated tumour cells (DTCs) when they enter bone marrow, where they form tumour reservoirs [6,7,8,9,10,11]. The existence of CTCs was already shown in 1869 by Thomas Ashworth [12], who found tumour cells in the blood of a person, who died of metastastic cancer. Nowadays it is known that the occurrence of CTCs in patients with epithelial cancer is linked to a worse prognosis [5,13]. Therefore the occurrence of CTCs in the blood stream (and DTCs in bone marrow) was included in the international tumour staging systems [14,15]. According to the 7th AJCC Breast Cancer Staining Manual, not only stages M0 and M1 account for metastatic stages of a tumour, but cM(i+) patients, who carry CTCs/DTC in blood or bone marrow without further clinical or radiographic evidence for metastases are described as metastases positive patients [16].

## 3. CTC-Detection Methods

The detection of DTCs from bone marrow is a non-standardized and time consuming procedure. In contrast, detection of CTCs from blood samples is much easier due to the access to suitable biomaterial. The only disadvantage of CTC-detection from peripheral blood is the small number of CTCs (1 per 10^6–7^ cells) [17]. Up until now there is only one FDA-approved system for CTC-detection, at least in the metastatic setting. The CellSearch^®^ system, distributed by Veridex LLC [18] is based on an immunomagnetic enrichment with following staining of epithelial cell surface markers.

Various other detection methods are known [19], most of which are based on an enrichment of tumour cells via the surface marker EpCAM. One example is the Adna-Test, followed by the auto MACS magnetic activated cell sorting, the CTC Chip technology, and the Dynabeads^®^ Epithelial Enrichment. Other methods are based on a depletion of normal blood or bone marrow cells by CD45. This method uses the Dynabeads^®^ CD45-technique. Another system is called OMS or the RosetteSep-Applied Imaging Rare Event (RARE) system. Furthermore, there are methods for CTC/DTC enrichment and isolation. These systems use different cell sizes (CTC membrane microfilter or ISET) or FAST Cytometry (FACS) [20]. A very recently described technique called “CTC-iChip” [21] is capable of sorting rare CTCs from whole blood, independently of tumour membrane epitopes. It seems to offer great promise for future CTC and DTC detection and characterization because isolated tumour cells are of high morphological quality and will allow a broad range of downstream analyses. In the future, studies with high numbers of patients will be necessary to prove the clinical use of all these newly developed techniques [22]. The research on CTCs could be prospectively supplemented by the upcoming field of cfDNA (cell free DNA) analyses, which might contribute new insights into cancer research [23,24].

## 4. Real-Time PCR

Another method for a molecular detection of CTCs is the highly sensitive Real-Time PCR. This method is exclusively used in the Adna-Test [25]. The test is based on the fact that CTCs are of epithelial origin. Therefore CTC express different gene panels compared to surrounding blood cells, which are of mesenchymal origin. Tumour cell positive blood samples contain certain genes that are less expressed in a sample without CTCs (negative reference sample).

Real-Time PCR is a variant of a PCR reaction, in which the amplification of a certain gene is measured by an increase in fluorescence in the exponential phase of PCR reaction. Reporter molecules are added to the PCR reaction, which render a fluorescent signal when incorporated into the newly synthesized DNA-molecules. Such a reporter molecule is the intercalating DNA stain Sybr Green [26]. In general, there are two methods of Real-Time PCR evaluation: absolute and relative quantification. For absolute quantification a standard curve has to be generated, which is a laborious, time consuming and an expensive procedure. In contrast, for relative quantification only a normalization of gene expression towards a reference gene has to be done. The reference gene is called housekeeping gene because it is ubiquitously expressed. Most used housekeeping genes are GAPDH or beta-actin [27].

If a target gene is highly expressed, the amount of amplified DNA-fragments will increase rapidly. The fluorescence signal increases rapidly too and reaches the threshold value sooner compared to a gene which does not have such a high expression. The number of PCR cycles when fluorescence extends the threshold is called Ct-value. A small Ct-value counts for a highly expressed gene. The Ct-value of a target gene is referred to a housekeeping gene, for example 18S or GAPDH, which are ubiquitously highly expressed. The resulting value is called ΔCt-value and is again set in reference to a control sample. Thereby the ΔΔCt-value is the basis for the calculation of the relative quantification (RQ) value by the formula published by Livak *et al.*: 2^−ΔΔCt^ [28]. If this value is >1 an examined gene is upregulated, if RQ-value is <1 the gene is downregulated in comparison to a certain reference sample.

An even more sensitive variant of Real-Time PCR is TaqMan PCR [29], which was invented by Cetus Corporation (Berkeley, CA, USA) in 1991. In TaqMan PCR, in additional to the primers for a specific gene another also gene specific probe is added to the reaction. This TaqMan or hydrolysis probe has two reporter molecules bound at the end. These are called reporter and quencher. If both reporter molecules are bound to the probe, no fluorescence signal is transmitted due to Fluorescence Resonance Energy Transfer (FRET). Other types of additional PCR probes working on FRET are for example Loop Tag-Probes, Molecular Beacons or Scorpion Primers. If the probe binds to the respective DNA sequence and the PCR amplification goes on, the quencher is released from the probe by the 5'–3' exonuclease activity of the polymerase. In consequence, the reporter molecule emits fluorescence, which is then recorded by the Real-Time PCR machine.

## 5. CTC-Detection via Real-Time PCR

For the application of Real-Time PCR in tumour cell detection from peripheral blood samples some tumor cell pre-enrichment strategies have to be applied. As a first step for tumour cell enrichment from blood samples enrichment via EPCAM could be done. Another way would be a simple enrichment of tumour cells by density gradient centrifugation. This procedure is applicable as CTCs have similar size and properties as leucocytes. Therefore the simple aspiration of red blood cells after the first centrifugation step leads to enriched tumour cells. In the next step, appropriate marker genes have to be selected. A good choice are for example cytokeratin (CK) genes [30], CK8, 18 or 19. These three genes are typical epithelial genes with only very low expression in leukocytes and high expression in tumour cells. Furthermore these genes are suitable because they are also used in the APAAP-staining, which is routinely used for immunhistochemical cancer cell detection [31,32]. CK19 has additionally been used in a large amount of studies concerning CTCs. It has been shown to be the most effective PCR marker gene for breast cancer, also in combination with various other marker genes. In 2007 and 2008, Ignatiadis *et al.* [33,34] found that the presence of CK19 mRNA, detected by Real-Time PCR predicted a poor clinical outcome in patients with ER-negative, triple negative or Her2 positive breast cancers. CK19 is a marker for shorter disease free survival and reduced overall survival in patients before adjuvant chemotherapy. Daskalaki *et al.* [35] examined the correlation of CK19 positive CTCs and DTCs and found that CTC detection by Real-Time PCR is not inferior to DTC detection methods. This is much more tolerable for the patients as no bone marrow has to be taken, which is a painful procedure. Recent studies verified that a multimarker gene panel for Real-Time PCR is a good choice to detect CTCs in all adenocarcinomas, especially in metastatic breast cancer [36,37]. It was found out, that patients with primary breast cancer having CK19 positive CTCs have a poor prognosis, independent of the treatment strategy [38]. Their appearance in patients with metastatic breast cancer defines a subgroup of patients with poor outcomes in general [39]. Furthermore, a study by Joosse *et al.* reportscomplex patterns of cytokeratin expression in breast carcinomas, changes in cytokeratin expression during metastatic progression, and an association of CK16 expression with shortened relapse-free survival in metastatic breast cancer patients [40]. Hence more research is needed in the field of cytokeratins and their role in breast cancer, as they seem to be promising marker genes.

The third step of CTC analysis would be the creation of calibration curves. Therefore, a certain amount of cells from mamma carcinoma cell lines is added to blood samples from healthy donors. Real-Time PCR is carried out as described and relative quantification (RQ)-values for all samples are measured. Calibration curves are generated from the RQ-values of blood samples spiked with increasing numbers of tumour cells [41]. The analysis of patient samples can be related to RQ-values of the calibration curves. Conclusions can be drawn to the number of CTCs in a patient sample [42].

However, the method of CTC detection by quantitative RT-PCR has some limitations [43]. First of all, normal epithelial cells derived from patient skin during withdrawal of peripheral blood could contaminate the blood sample. This would lead to false positive results. Furthermore, during density gradient centrifugation of the blood sample some CTCs might get lost in the procedure. The next step, isolation of RNA from the harvested cells, is one of the most critical steps in the whole process preparing RT-PCR, as RNases are ubiquitous and RNA could be degraded rapidly. In the following reverse transcription reaction an amplification of processed pseudogenes could occur, thereby falsifying results of RT-PCR. The major challenge in Real-Time PCR is to keep reaction conditions, such as ion concentrations, amounts of primers and dNTPs and Taq-Polymerase constant over all reactions. These drawbacks can at least in part be overcome by the performance of adequate control reactions. For example a denaturing gel approach could be applied to check RNA degradation. Nevertheless, one obstacle still remains: sometimes a basal expression of epithelial cell genes is also found in normal hematopoietic cells. The expression of epithelial genes in CTCs can vary severely. Therefore, it is indispensable to carry out PCR-reactions with negative control samples simultaneously.

## 6. Conclusions and Future Prospective

Real-Time PCR had been used for tumour cell detection and analysis in material from solid tumours [44,45] and even in some forms of leukemia [46,47]. This method is furthermore used in HPV detection of head and neck squamous cell carcinoma [48]. Real-Time PCR is highly sensitive and gene-specific and in addition, cost-effective and robust. Real-Time PCR has additionally been shown, to be a rather sensitive technique for CTC detection. The comparison of the AdnaTest and the CellSearch^®^ system in respect to the prognostic relevance of CTC detection, it is still controversial which method is superior [49,50]. It is necessary to further improve the method for an application in the *in vivo* situation. In such a situation, gene expression levels vary between patients [51,52]. Therefore, more marker genes like for example MMP13 [53], UBE2Q2 [54], Nectin-4 [55] or ALDH [56] will have to be tested for their ability not only in tumour cell detection but also in tumour cell characterization. This characterization could give hints towards cancer prognosis. For example, Bölke *et al.* described in 2009 [57], that the expression of certain genes is related to advanced breast cancer stages. Additionally, a characterization of CTCs could help to personalize treatment by increasing efficiency and reducing side effects, resulting in a better outcome and an increased survival, rendering great benefit to the patients.

The wide field of DNA methylation profiling [58], which is crucial for gene silencing and activation, by PCR based approaches has already been described and will certainly gain importance in the next time. Another challenging task for the future will be to combine methods like Real-Time PCR with next generation sequencing methods (NGS) [59,60]. This will lead to a more precisely analysis of tumour samples and to a determination of prognostic ability and treatment benefit based on these new methods [61].
